# Research on Cold Chain Logistics Joint Distribution Vehicle Routing Optimization Based on Uncertainty Entropy and Time-Varying Network

**DOI:** 10.3390/e27050540

**Published:** 2025-05-20

**Authors:** Huaixia Shi, Yu Hong, Qinglei Zhang, Jiyun Qin

**Affiliations:** 1 Logistics Engineering College, Shanghai Maritime University, Shanghai 201306, Chinaelmerh@foxmail.com (Y.H.); jyqin@shmtu.edu.cn (J.Q.); 2 Business School, Shanghai DianJi University, Shanghai 201306, China

**Keywords:** vehicle routing problem, joint distribution, cold chain logistics, improved genetic algorithm

## Abstract

The sharing economy is an inevitable trend in cold chain logistics. Most cold chain logistics enterprises are small and operate independently, with limited collaboration. Joint distribution is key to integrating cold chain logistics and the sharing economy. It aims to share logistics resources, provide collective customer service, and optimize distribution routes. However, existing studies have overlooked uncertainty factors in joint distribution optimization. To address this, we propose the Cold Chain Logistics Joint Distribution Vehicle Routing Problem with Time-Varying Network (CCLJDVRP-TVN). This model integrates traffic congestion uncertainty and constructs a time-varying network to reflect real-world conditions. The solution combines simulated annealing strategies with genetic algorithms. It also uses the entropy mechanism to optimize uncertainties, improving global search performance. The method was applied to optimize vehicle routing for three cold chain logistics companies in Beijing. The results show a reduction in logistics costs by 18.3%, carbon emissions by 15.8%, and fleet size by 12.5%. It also effectively addresses the impact of congestion and uncertainty on distribution. This study offers valuable theoretical support for optimizing joint distribution and managing uncertainties in cold chain logistics.

## 1. Introduction

Economic advancement has increased living standards and raised demand for cold chain products. This surge has been driven by e-commerce and cold chain logistics companies. The Asia Pacific region, particularly China, has benefited the most. Despite the rapid growth of China, challenges hinder the cold chain logistics industry [1]. Many logistics companies are small, offering single distribution services [2]. Cold chain products are perishable and require costly refrigerated transportation. Other challenges include small order batches, diverse customer locations, strict timelines, low vehicle load rates, and high carbon emissions [3]. To overcome these obstacles, strategic distribution adjustments are needed.

The vehicle routing problem (VRP), a standard model in route planning, aims to decrease transportation cost and distances by route optimization [4]. Cold chain logistics necessitate additional fuel to maintain product freshness, meaning damage, refrigeration, and carbon emissions factors need to be accounted for [5]. Unfortunately, there is limited literature addressing these costs. Furthermore, the dynamic nature of urban distribution, affected by traffic congestion and changing vehicle statuses, clashes with traditional static optimization methods [6].

Many studies have explored the VRP to optimize vehicle routes in cold chain logistics [7,8,9]. However, these studies predominantly focus on single distribution and often overlook the benefits of synchronizing operations. Joint distribution refers to multiple logistics companies sharing resources and customers under a unified plan [10]. This approach can significantly enhance efficiency, reduce losses, and lower overall distribution costs [11].

Many studies have addressed vehicle route optimization in cold chain logistics using the vehicle routing problem (VRP), creating models based on cold chain features. Hsu et al. initially proposed a VRP model for fresh food logistics with time windows, factoring in the stochastic nature of perishable fresh food [12]. Further, Osvald et al. integrated multiple costs and established a vehicle route planning model for perishable fresh food logistics distribution [13]. Meanwhile, Sun et al. incorporated time windows into their cold chain logistics model to ensure product quality and enhance customer satisfaction [14]. Amorim et al. developed a multi-time window, multi-model vehicle routing problem for food distribution in Portugal [15].

Though aiming to reduce cargo damage, maintain freshness, and improve satisfaction, previous studies overlooked refrigeration and carbon emission costs in cold chain transport. Zhang et al. introduced a low-carbon economy to cold chain logistics, developing an optimization model that includes carbon emission costs [16]. Qin et al. studied the impact of carbon pricing on emissions and customer satisfaction [17]. Wang et al.’s model considered damage costs during loading and unloading but ignored delivery costs [5]. Previous studies frequently overlooked comprehensive cost components. The present study addresses this gap by considering cargo damage, refrigeration, time penalty, and carbon emission costs.

Many researchers have examined the VRP problem concerning vehicle speed variation. Davis modelled traffic congestion based on roadway vehicle numbers, targeting travel time minimization [18]. Wang et al. predicted traffic flow using a Kalman filter and developed a dynamic path model that prioritized travel time minimization and road capacity constraints [19]. Taniguchi et al. used advanced intelligent transportation systems for urban distribution, creating a vehicle path model that updates with real-time travel time changes using traffic simulation data [20]. Liao et al. proposed a two-stage method for time-dependent travel times, using a sweep method for vehicle assignment and a forbidden search algorithm for path enhancement [21]. Sabar et al. considered various road traffic conditions in their DVRP model, minimizing total distribution costs, and introduced an adaptive evolutionary algorithm [22]. Kim et al. addressed time-varying traffic flow, aiming for total cost and vehicle count minimization, and established a VRP model with time-window constraints [23]. Güner et al. differentiated periodic and non-periodic congestion, using a random queuing model for time delay estimation and setting travel time minimization as the objective [24]. Many studies on vehicle routing problems with time-varying networks exist. However, few have considered real-time traffic information in cold chain logistics. Given the urban distribution and time-sensitive nature of cold chain products, further research is necessary.

Currently, many cold chain logistics companies use single distribution. Although widely studied, this approach has drawbacks such as poor fill rates, extensive vehicle leasing, and high delivery costs. These issues considerably hamper industry progress. Joint distribution, aimed at enhancing efficiency through resource integration, originated in Japan. It enables resource sharing and significantly reduces operation costs [10].

Resource sharing is key to efficient vehicle routing in joint distribution, leading to lower logistics costs and improved customer satisfaction. Wang et al. developed an open multi-center joint distribution model based on vehicle sharing [25]. Wang and Zhang constructed an MDVRP model integrating set and delivery, using a hybrid heuristic algorithm and logistics center and customer information sharing [26]. Zhang and Chen designed a joint distribution model with shared logistics centers and vehicles, solved using a multi-objective optimization model and variable neighborhood search algorithm [27]. Ramachandran compared inventory and distribution costs under joint and direct strategies, showing a clear truck load rate advantage for joint distribution [28]. Li et al. proposed a hybrid genetic algorithm with adaptive neighborhood search for a joint distribution problem, considering shared parking lot resources and fuel consumption [29].

Although numerous studies have addressed either the specific cost structure of cold-chain logistics, the dynamics of time-varying road networks, or the advantages of joint distribution, very few works capture all three aspects simultaneously. Table 1 summarizes the most relevant publications and highlights the missing combinations that motivate the present study.

Despite existing joint distribution research, its application in cold chain logistics remains underexplored. This study introduces the vehicle routing problem of cold chain logistics joint distribution with time-varying networks. Based on previous studies, three gaps can be identified: (1) the cost structure of cold chain logistics is incomplete, despite higher distribution requirements and more complex costs compared to general logistics; (2) existing studies often assume constant traffic conditions, neglecting time-varying transportation factors; (3) research primarily emphasizes economic benefits of joint distribution, rarely focusing on path optimization for cold chain logistics vehicles.

In order to address these research gaps, this study proposes a new model: the Cold Chain Logistics Joint Distribution Vehicle Routing Problem with Time-Varying Networks (CCLJDVRP-TVN). The model considers cold chain logistics costs, integrates sharing economy principles, and captures time-varying road network dynamics. An improved genetic algorithm with simulated annealing strategies is designed to solve the CCLJDVRP-TVN problem. Genetic algorithms often prematurely converge to local optima. To overcome this, the method introduces adaptive crossover and mutation probabilities and incorporates simulated annealing strategies. These enhancements improve global search, solution quality, convergence speed, and stability, demonstrating superior performance in complex cold chain logistics vehicle routing.

## 2. Cold Chain Logistics Joint Distribution Vehicle Routing Model

This section clarifies the CCLJDVRP-TVN problem setting, defines the supply-chain entities and key operational assumptions, and introduces the notation used in the subsequent model.

### 2.1. Problem Description and Assumption

**Figure 1:**
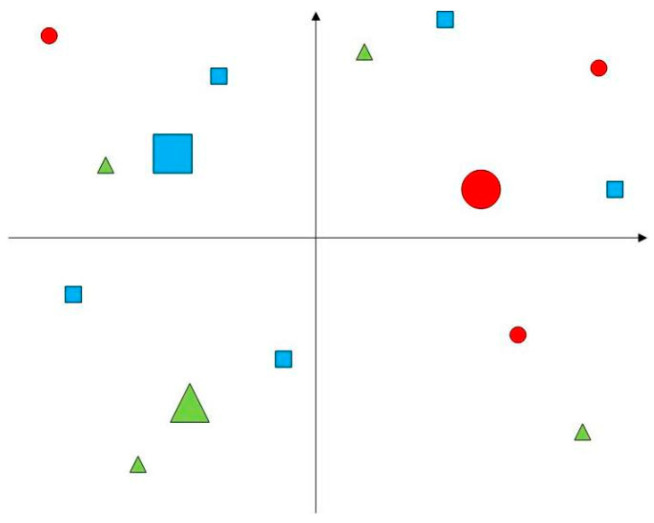
Regional depots and customers.

**Figure 2:**
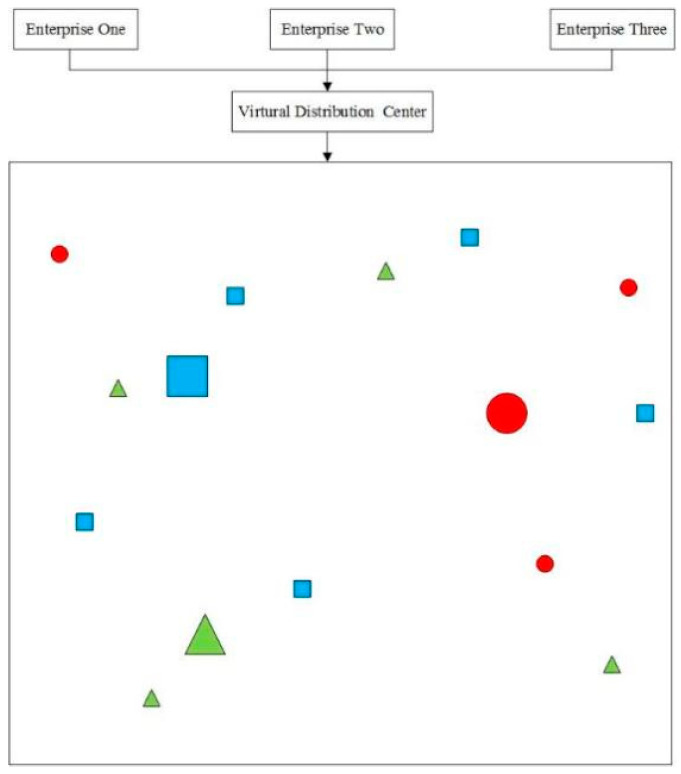
Joint distribution mode.

**Figure 3:**
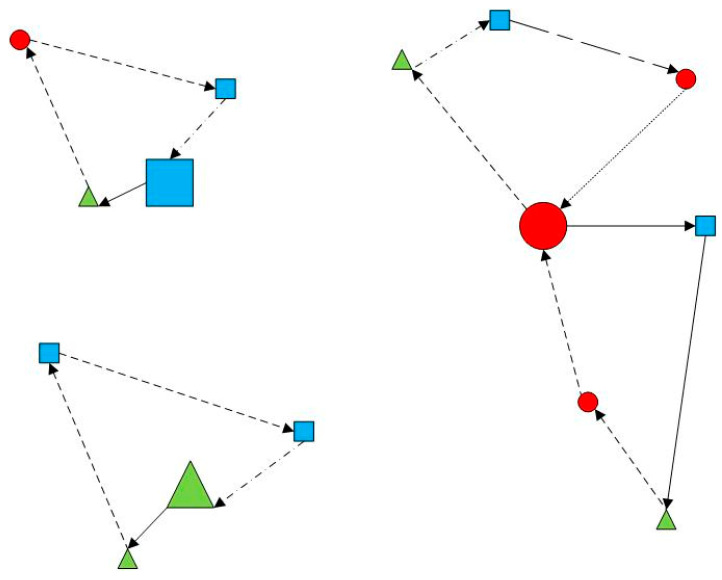
Vehicles travel at different speeds.

This study examines the joint distribution vehicle routing problem in cold chain logistics, considering time-varying networks. Multiple cold chain distribution centers serve different customers (Figure 1). Under joint distribution, all centers share storage and vehicle resources to serve all customers (Figure 2). Goods demand, time windows, and locations are known at customer points. City traffic congestion affecting vehicle speed is represented (Figure 3). In Figure 1, Figure 2 and Figure 3, red circles represent centers, blue triangles represent customer locations, green lines indicate routes, and black dashed lines denote time-window constraints. Colors and symbols represent different nodes and their connections. Perishable cold chain products require refrigerated transport, incurring damage, refrigeration, and carbon emission costs. The study aims to find the optimal vehicle scheduling and routing solution, considering these costs, vehicle transport, overtime penalty, and usage.

The following assumptions are made for analysis and research:

1. Known locations of all centers and customers. Vehicles depart from any center, return to the nearest after delivery, no replenishment.
2. Identical refrigeration vehicles used, consuming fuel for transport refrigeration.
3. Refrigeration vehicles runs while waiting and serving at customers. More fuel used when doors open for cooling.
4. Vehicles avoid overloading, varied speeds due to traffic.
5. Cargo demand and time windows set for each customer. Vehicles arrive early or late, incurring penalties.
6. Each customer served by one vehicle with cargo ≤ capacity.

### 2.2. Symbols and Variables

The mathematical model of the model is established as follows: assume that M is the set of distribution points, N is the set of customer points, V is the total set, and V = M∪N = {1, 2, …, *m*, *m* + 1, *m* + 2, …, *m* + *n*}, where *n* is the number of customer points and {1, 2, …, *m*} is the distribution centers. The relevant variables and parameters of the model are shown in Table 2.

### 2.3. Multi-Logistics Centers Joint Distribution Processing Method

For resource sharing, a virtual distribution center C0 is added, linked to all centers. Vehicles start from C0, deliver to centers, return, and end at C0. Travel time between C0 and centers is 0.

### 2.4. Vehicle Travel Time Calculation

**Figure 4:**
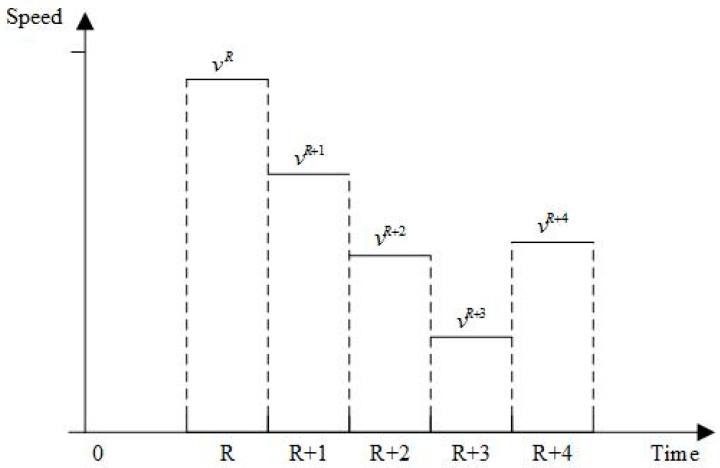
Vehicle speed in different time periods.

**Figure 5:**
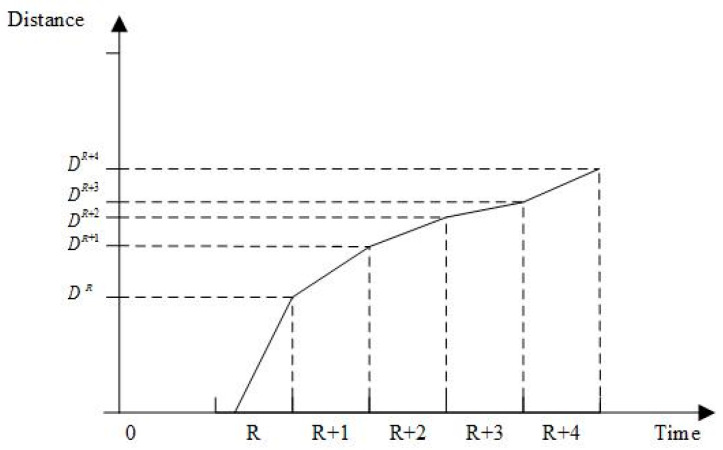
Driving distance in different time periods.

Distribution vehicles’ speeds vary due to traffic, resulting in time-varying behavior. Some road sections may require detours due to traffic conditions, complicating travel time calculations. The interplay between time periods, vehicle speeds, distances, and travel times are examined. Vehicle speeds and distances in distinct time periods are shown in Figure 4 and Figure 5.

Let T be the length of the working time period of the distribution center, and divide T into z time periods $\left\{T_{1},T_{2},T_{3},...,T_{z+1}\right\}$, where $\left[T_{R},T_{R+1}\right]$ is the R time period and $v^{R}$ represents the vehicle travel speed in time period R. The $v\left(t_{i}\right)$ represents the velocity step function; when $t_{i}\in \left[T_{R},T_{R+1}\right]$, $v\left(t_{i}\right)=v^{R}$.

Assuming that $t_{i}$ is the time when the vehicle leaves node i, a vehicle traveling on path $\left(i,j\right)$ will have two cases:

1. The vehicle is in one time period from i to j, so that the vehicle has only one speed on this path.
2. The vehicle spans two and more time periods from i to j, so that the vehicle has two and more speeds on this path.

According to the above characteristics, it is necessary to calculate the time from i to j based on the vehicle speed of the time period. S=T/z denotes the length of the time interval in which each speed is constant in the speed step function, $t_{ex}$ denotes the time interval between moment t and the next speed change, $d_{ex}$ denotes the length that can be traveled according to the speed of moment t for the length of time of driving $t_{ex}$, and $T_{i,j}\left(d_{ij},t_{i}\right)$ denotes the running time of the vehicle from i to j. The travelling time from i to j is calculated as follows:

(1) $$T_{i,j}\left(d_{ij},t_{i}\right)=\left\{\begin{array}{c} t_{ex}+T_{i,j}\left(d_{ij}-d_{ex},t_{i}+t_{ex}\right)\;\;\;,d_{ij}-d_{ex}>0\\ \frac{d_{ij}}{v\left(t_{i}\right)}\;\;\;\;\;\;\;\;\;\;\;\;\;\;\;\;\;\;\;\;\;\;\;\;\;\;\;,else \end{array}\right.$$

where

$$t_{ex}=\left\{\begin{array}{c} \left|\frac{t}{s}\right|s-t\;\;,\left|\frac{t}{s}\right|>0\\ s\;\;\;\;\;\;\;\;\;\;,else \end{array}\right.$$

$$d_{ex}=t_{ex}v\left(t_{i}\right)$$

Equation (1) indicates that when the vehicle is traveling in the first case, the travelling time is the ratio of the distance to the speed in the current time period. When the vehicle is the second case, the vehicle departs from i and has a distance of $d_{ij}$ from j at time t. If the total length of driving at the speed of time t to the next speed change moment, $d_{ex}<d_{ij}$, then it needs to enter the next time period to continue driving, and the time length from time *t* to reach point j is the sum of $t_{ex}$ and $T_{i,j}\left(d_{ij}-d_{ex},t_{i}+t_{ex}\right)$.

### 2.5. Cost Variables

This research aims to minimize the total distribution cost of cold chain logistics, considering comprehensive supply chain costs to reflect the actual distribution process. The total cost of CCLJDVRP-TVN includes: (a) fixed costs, (b) transportation costs of refrigeration trucks, (c) penalties for delivery time non-compliance, (d) damage costs from partial goods damage, (e) refrigeration costs in low-temperature environments, and (f) carbon emission costs from refrigeration trucks and equipment. Each cost component is explained below.

1. Fixed cost *C*1:

The fixed cost of vehicles includes depreciation, driver labor, and vehicle rent. It does not change with customer numbers or distribution distances, only with vehicle usage. The fixed cost is calculated as follows:

(2) $$C1=K\cdot C_{f}$$

where $C_{f}$ denotes the fixed cost of the vehicle and *K* denotes the number of vehicles in use.

2. Transportation cost *C*2:

Vehicle transportation cost includes fuel consumption during distribution. It is positively related to the distance traveled by the vehicle. The transportation cost is given by:

(3) $$C2=C_{t}\sum_{i=1}^{m+n}\sum_{j=1}^{m+n}\sum_{k=1}^{K}x_{ijk}d_{ij}$$

where $C_{t}$ indicates the transportation cost per unit distance, $d_{ij}$ indicates the distance from point i to point *j*, and $x_{ijk}$ is a 0–1 variable. When vehicle k travels from point *i* to point *j*, the value is 1; otherwise, the value is 0.

3. Time Penalty Cost *C*3:

In cold chain logistics, timely product delivery is crucial as it affects revenue, inventory control, and quality management. The vehicle must meet the customer’s time window. If the vehicle arrives too early, it must wait; if too late, restocking and sales issues may arise. Penalty costs occur if the vehicle misses the time window [30,31]. This paper uses a soft time window with a penalty function to represent the cost, as shown in Equation (4):

(4) $$C3=\sum_{m+1}^{m+n}\sum_{k=1}^{K}\left[C_{e}\max\left(T_{e}-T_{jk},0\right)+C_{l}max\left(T_{jk}-T_{l},0\right)\right]$$

where $C_{e}$ and $C_{l}$ denote the penalty costs due to early arrival and late arrival, [$T_{e},T_{l}$] denotes the time window required by the customer, and $T_{jk}$ denotes the time when vehicle *k* arrives at customer point *j*.

4. Damage cost *C*4:

During the distribution process, the quality of the cold chain products deteriorates to some extent due to time, which results in damage costs [32,33]. Quality loss is shown as an exponential change with the advance of time, and the damage cost is calculated as follows:

(5) $$C4=C_{p}\cdot \sum_{i=1}^{n}g_{i}\cdot \varepsilon (1-e^{-\theta W_{i}})$$

where $C_{p}$ is the price per unit of product, $g_{i}$ is the customer demand at point *i, ε* is the deterioration rate of the product during transportation and loading and unloading, *θ* is the product sensitivity coefficient, and $W_{i}$ is the time from the departure of the vehicle to point *i*.

5. Refrigeration cost *C*5:

Cold chain logistics vehicles require refrigeration equipment powered by the engine to maintain low temperatures, generating refrigeration costs [7,34]. This paper assumes that opening and closing doors does not affect the temperature difference, and carbon emissions from temperature changes are not considered.

(6) $$C51=C_{r}\cdot \alpha_{1}\cdot \sum_{i=1}^{m+n}\sum_{j=1}^{m+n}\sum_{k=1}^{K}x_{ijk}\cdot t_{ij}$$

(7) $$C52=C_{r}\cdot \alpha_{2}\cdot \sum_{i=1}^{m+n}\sum_{j=1}^{m+n}\sum_{k=1}^{K}x_{ijk}\cdot S_{j}$$

*C*5 = *C*51 + *C*52

where $C_{t}$ denotes the unit fuel price, $\alpha_{1}$ denotes the fuel consumption of refrigeration equipment per unit time of vehicle travel, and $\alpha_{2}$ denotes the fuel consumption generated by the refrigeration equipment during the loading and unloading process per unit time. $t_{ij}$ denotes the time of vehicle from node *i* to *j*, $S_{j}$ denotes the service time of customer *j*, and $x_{ijk}$ is a 0–1 variable with a value of 1 when vehicle k travels from point *i* to point *j*, and 0 otherwise.

6. Carbon emissions cost *C*6:

During transportation, vehicle fuel consumption generates carbon dioxide, contributing to the greenhouse effect. Reducing carbon emission costs lowers distribution costs and environmental harm [35]. Carbon emissions mainly come from fuel consumption, which is related to vehicle driving time.

For the purposes of this paper, refrigeration equipment generates carbon emissions.

$$FC1=\alpha_{1}\cdot \sum_{i=1}^{m+n}\sum_{j=1}^{m+n}\sum_{k=1}^{K}x_{ijk}\cdot t_{ij}$$

$$FC2=\alpha_{2}\cdot \sum_{i=1}^{m+n}\sum_{j=1}^{m+n}\sum_{k=1}^{K}x_{ijk}\cdot S_{j}$$

Also, according to Zhang and other scholars [36], carbon emissions due to vehicle load during distribution:

$$FC3=\beta \cdot \sum_{i=1}^{m+n}\sum_{j=1}^{m+n}\sum_{k=1}^{K}x_{ijk}\cdot d_{ij}\cdot Q_{ij}$$

where *β* denotes the load carbon emission factor and $Q_{ij}$ denotes the load between point i and point *j* of the section.

The carbon emission is the product of fuel consumption and ${CO}_{2}$ emission factor. Thus, the carbon emission can be expressed as:

$$EM=\eta \cdot \left(FC1+FC2+FC3\right)$$

The cost of carbon emissions can be expressed as:

(8) $$C6=C_{c}\cdot \eta \cdot \sum_{i=1}^{m+n}\sum_{j=1}^{m+n}\sum_{k=1}^{K}x_{ijk}\left[\alpha_{1}\cdot t_{ij}+\alpha_{2}\cdot \left(t_{ij}+S_{j}\right)+\beta \cdot d_{ij}\cdot Q_{ij}\right]$$

### 2.6. Modeling

The total cost of cold chain transportation includes fixed cost (*C*1), transportation cost (*C*2), penalty cost (*C*3), damage costs (*C*4), fuel consumption cost (*C*5), and carbon emission cost (*C*6). Thus, the mathematical model is expressed as follows.

(9) $$minC=C_{1}+C_{2}+C_{3}+C_{4}+C_{5}+C_{6}$$

(10) $${\;\;\;\;\;\;\;\;\;W}_{j}=W_{i}+S_{i}+t_{ij},i\neq j,\;i\in V,j\in V,W_{0}=T_{s}$$

(11) $$s.t.\sum_{i=m+1}^{m+n}x_{ijk}=1,j\in \left\{m+1,m+2,\ldots ,m+n\right\}$$

(12) $$\;\;\;\;\;\;\;\sum_{j=m+1}^{m+n}x_{ijk}=1,j\in \left\{m+1,m+2,\ldots ,m+n\right\}$$

(13) $$\;\;\;\sum_{j=m+1}^{m+n}\sum_{i=1}^{m}\sum_{k=1}^{K}x_{ijk}=\sum_{j=m+1}^{m+n}\sum_{i=1}^{m}\sum_{k=1}^{K}x_{jik}\leq 1$$

(14) $${\;\;\;\;\;\;\;\;\;\;\;Q}_{ij}\leq Q,i\in \left\{1,2,\ldots ,m\right\}\;$$

(15) $$\;g_{i}\leq Q,i\in \left\{m+1,m+2,\ldots ,m+n\right\}$$

(16) $$\;\sum_{i=1}^{m}x_{ijk}=0,i\in \left\{1,2,\ldots ,m\right\}$$

(17) $${\;\;\;\;\;\;\;\;\;x}_{ijk}=\left\{0,1\right\}$$

Equation (9) minimizes distribution costs. Equation (10) relates vehicle arrival time, transportation time, service time, and start time. Equations (11) and (12) ensure each customer is visited once. Equation (13) allows the vehicle to return to a different distribution center after completing the task. Equations (14) and (15) impose capacity constraints, ensuring the vehicle is not overloaded and customer demand is met. Equation (16) ensures vehicles do not visit other distribution centers for replenishment before completing the task. Equation (17) sets value constraints for decision variables.

## 3. Model Solution

Genetic algorithms have been used to solve VRP, but traditional methods converge slowly and are prone to local optima. To overcome this, we propose an improved genetic algorithm (IGA) integrated with simulated annealing. The IGA combines simulated annealing’s global exploration with adaptive crossover and mutation probabilities. This improves convergence speed, solution quality, and global search capabilities, addressing the limitations of traditional genetic algorithms in modern VRP research.

### 3.1. Algorithm Initialization

#### 3.1.1. Chromosome Coding

**Figure 6:**
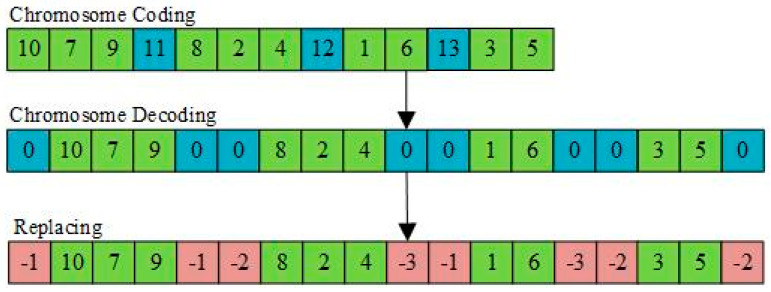
Chromosome coding and decoding.

In this study, integer encoding is used, with negative numbers {−*m*, −*m* − 1, …, −1} denoting distribution centers, positive integer numbers {1, 2, …, *n*} denoting customer points, and {*n* + 1, *n* + 2, …, *n + k*} denotes the number of available vehicles. The length of the chromosome is *n + k* − 1. The decoding process of chromosomes is shown in Figure 6. Taking 10 customer locations, four available vehicles and four distribution centers as an example, the virtual distribution center 0 is first inserted at the first end of the chromosome, and then the number representing the vehicle in the chromosome {*n* + 1, *n* + 2, …, *n + k*} is replaced with virtual distribution center 0. After traversing all chromosomes, 0 is replaced with the nearest distribution center to the customer point according to the customer point to the left or right of virtual distribution center 0.

#### 3.1.2. Population Initialization

Using random generation, popsize individuals form the initial population pop(h). The initialization iteration number is h = 1, T(1) is the initial temperature, and Max is the maximum iterations. If T(1) is too large, the probability of infeasible solutions increases, resulting in longer iterations and difficulty in convergence. If T(1) is too small, the probability of infeasible solutions decreases, leading to local optima. The simulated annealing algorithm is used to address this issue in this study.

#### 3.1.3. Fitness Function

The fitness of each chromosome in the population can be constructed by the model objective function Equation (18). In this study, the objective function value is the total distribution cost, so the smaller the distribution cost, the larger the chromosome fitness value, so the reciprocal of the objective function is selected as the fitness degree. Through the calculation of this fitness function, it is possible to determine whether the solution is feasible. If it is not feasible, a penalty value is added.

(18) $$f_{i}=\frac{1}{C}$$

### 3.2. Algorithm Operator

#### 3.2.1. Selection Operation

Chromosomes are selected in the overall by roulette selection method. The higher the chromosome fitness, the higher the probability of roulette selection, as shown in Equation (19). $p_{i}$ is the probability that the individual i is selected, $f_{i}$ and $f_{j}$ are the fitness values of the individual i and j, and popsize is the population size. $p_{0}=0$, $R_{1}$ is a random number in the interval [0, 1], and individual i is selected when $\sum_{j=0}^{i-1}p_{j}\leq R_{1}\leq \sum_{j=0}^{i}p_{j}$. New population Newpop(h+1) is formed by cyclic selection through the selection operation.

(19) $$p_{i}=\frac{f_{i}}{\sum_{j=1}^{popsize}f_{j}}$$

#### 3.2.2. Cross-Mutation Operation

**Figure 7:**
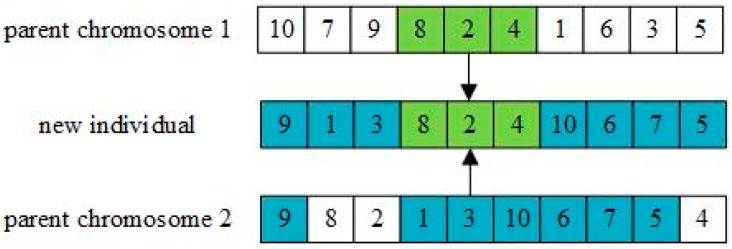
Order crossover operation.

The order crossover method is applied to Newpop(h) with crossover probability $P_{c}$ to obtain the new population Cpop(h). Order crossover is shown in Figure 7. Randomly select the start and end positions of several genes in a pair of parent chromosomes. Generate a new individual and ensure that the position of the selected genes in the new individual is the same as that of the parent, place one of the parent’s selected genes into the same position in the offspring, and place the other parent’s remaining genes into the offspring in order.

**Figure 8:**
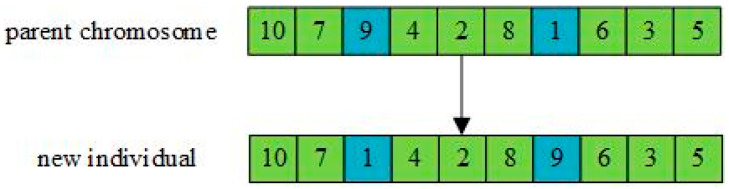
Mutation operation.

The population Cpop(h) is mutated with mutation probability $P_{m}$ to obtain a new population Mpop(h). The mutation operation is shown in Figure 8, where two positions on the parent chromosome are randomly selected and the genes at these two positions are exchanged to generate offspring.

Genetic operator probabilities (crossover and mutation) impact algorithm outcomes. For unfit individuals, increase crossover probability; for well-adapted individuals, reduce it. Thus, to sustain good candidates and avoid local convergence, this study applies adaptive crossover and mutation probabilities, as expressed in Equations (20) and (21).

(20) $$P_{c}=\left\{\begin{array}{c} P_{c1}-\frac{\left(P_{c1}-P_{c2}\right)\left(f^{\prime }-F_{ave}\right)}{F_{max}-F_{ave}},f^{\prime }\geq F_{ave}\\ \;\;\;\;\;\;P_{c1},f^{\prime }<F_{ave} \end{array}\right.$$

(21) $$P_{m}=\left\{\begin{array}{c} P_{m1}-\frac{\left(P_{m1}-P_{m2}\right)\left(F_{max}-f\right)}{F_{max}-F_{ave}},\;f\geq F_{ave}\\ P_{m1},\;f<F_{ave} \end{array}\right.$$

where $P_{c}$ refers to the crossover probability and $P_{m}$ refers to the mutation probability, $P_{c1}$ and $P_{c2}$ are the maximum and minimum values of the crossover probability, respectively, $P_{m1}$ and $P_{m2}$ are the maximum and minimum values of the mutation probability, respectively, $F_{ave}$ refers to the average fitness of the population, $F_{max}$ represents the maximum fitness of the population, $f^{\prime }$ is the larger adaptation value among the individuals to be crossed, and f is the adaptation value of the mutation to be made, where the adaptation of the individuals to be mutated is larger and the mutation probability is chosen to be lower. When the fitness value is lower, the population mutation and crossover probability values are larger, and the population diversity can be increased to avoid the algorithm from falling into local optimal solutions and to improve the global search ability.

#### 3.2.3. Simulated Annealing Improvement Operation

In early search stages, the genetic algorithm performs global exploration of the solution space. In later iterations, the solution set narrows, and local search weakens, hindering optimal solutions. To improve local search, a local random search is integrated. The simulated annealing algorithm processes infeasible solutions using the Metropolis criterion. Infeasible solutions are accepted based on temperature probability, as calculated in Equation (18), ensuring they are penalized and not propagated.

Simulated annealing is a randomized optimization method excelling in local search. Using the Metropolis criterion, new solutions can be accepted with certain probability, even if inferior, to escape local optima traps. Hence, this study adopts a local random search based on simulated annealing principles. Simulated annealing’s Metropolis criterion guides accepting new solutions during the search dominated by the original solution. The acceptance probability of new solutions is determined by:

(22) $$P_{s}=\left\{\begin{array}{c} e^{\left[-\left(f\left(x1\right)-f\left(x0\right)\right)/T\left(b\right)\right]},f\left(x1\right)-f\left(x0\right)<0\\ \;\;\;\;\;\;\;\;\;\;\;\;\;\;\;\;1\;\;\;\;\;\;\;\;\;\;\;\;\;\;\;\;\;\;,f\left(x1\right)-f\left(x0\right)\geq 0 \end{array}\right.$$

where $f\left(x1\right)$ is the adaptation value of the new solution, $f\left(x0\right)$ is the adaptation value of the original solution, and $T\left(b\right)$ is the temperature of the evolutionary generation. From the above equation, we can see that the acceptance probability $P_{s}$ is related to the temperature $T\left(b\right)$; the higher the temperature $T\left(b\right)$, the higher the acceptance probability of the dominated solution, while the lower the temperature $T\left(b\right)$, the lower the acceptance probability of the dominated solution. The iterative formula for the temperature $T\left(b\right)$ is:

(23) $$T\left(b+1\right)=\gamma T\left(b\right)$$

where γ is the cooling rate. The value of *T* decreases with increasing iterations, retaining potential solutions early on, enhancing solution space probability and global search. In later iterations, lower temperature reduces dominated solution acceptance probability, ensuring better solutions are preserved. In the present study, the initial temperature of the simulated annealing algorithm was set to 3000, and the cooling rate *γ* was 0.95.

**Figure 9:**
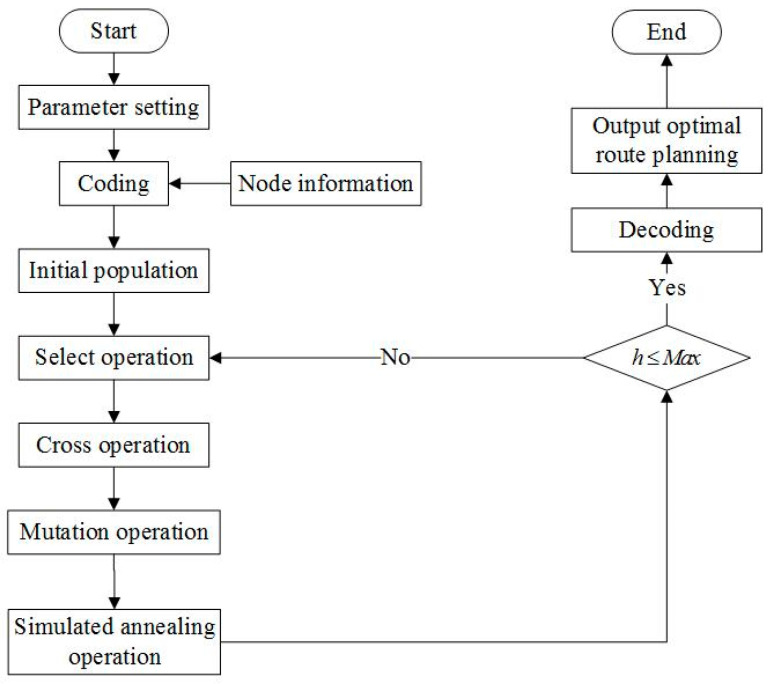
Flow chart of simulated annealing improved genetic algorithm.

The flowchart of the simulated annealing improved genetic algorithm proposed in the present study is shown in Figure 9. The pseudocode is given as in Algorithm 1:
**Algorithm 1.** Simulated Annealing Improved Genetic Algorithm**Input**:     Initial temperature T(1),     Max iterations (Max),     Neighborhood search limit (L),     Initial population size (popsize). **Output:**     Optimal vehicle routing solution (minimal total cost). 1: Initialize temperature T ← T(1) 2: Initialize iteration counter h ← 1 3: Generate initial population pop(h) randomly. 4: Evaluate fitness of each chromosome in pop(h). 5: WHILE h ≤ Max DO 6:        Perform selection operation to form new population pop’(h) 7:        Perform crossover and mutation operations with adaptive probabilities to form pop’’(h) 8:        Perform simulated annealing-based local random search:                 FOR each individual Xi in pop’’(h) DO                        SET neighborhood search count l ← 0                         WHILE l < L DO                                Generate new solution Yi near Xi                                ΔE ← fitness(Yi)–fitness(Xi)                                IF ΔE < 0 THEN                                       Accept Yi as new Xi; BREAK                                ELSE                                       Compute acceptance probability Ps = exp(−ΔE/T)                                       Generate random number rand ∈ [0, 1]                                       IF rand < Ps THEN                                              Accept Yi as new Xi; BREAK                                       ELSE                                              l ← l + 1                                       END IF                                 END IF                         END WHILE                 END FOR 9:          Update temperature T ← γT (γ = cooling rate) 10:      Update population pop(h) ← pop’’(h) 11:      h ← h + 1 12: END WHILE 13: RETURN optimal solution from final population.

The IGA begins by initializing the temperature, iteration counter, and population size. In each iteration, the algorithm evaluates chromosome fitness, performs selection, and applies crossover and mutation with adaptive probabilities. Simulated annealing performs a local random search, evaluating each solution and replacing it based on fitness difference. If the new solution improves fitness or is accepted probabilistically, it becomes the new solution. After each iteration, the temperature is updated, and the population is refined. The process continues until the maximum iterations are reached, returning the optimal solution.

## 4. Experimental Design and Analysis of the Results

In the present study, we validate the model using data from three Beijing cold-chain companies, compare independent versus joint-distribution scenarios, and perform congestion-sensitivity analyses.

### 4.1. Classical Dataset Test

Experiments were conducted with six MDVRP test cases (Pr01-Pr06) to test the improved genetic algorithm’s performance for the vehicle routing problem [37]. The test data can be accessed at: https://github.com/HuaixiaSHI/DATA. Both the basic and improved genetic algorithms were used to solve the dataset 20 times. The results are shown in Table 3.

Table 3 shows that the IGA’s mean shortest distance for each dataset is less than the ACO. The IGA’s optimal values are 0.63% (1082.35), 2.39% (1763.07), 6.93% (2408.42), 10.41% (2852.29), 13.61% (3029.65), and 1.98% (3758.36) lower than the ACO, with mean values lower than 4.11% (1086.21), 2.79% (1859.82), 7.79% (2501.01), 15.81% (2902.45), 10.71% (3388.55), and 4.95% (3870.85), respectively. Results show that the IGA outperforms the basic GA in optimization ability after adding adaptive crossover-mutation and simulated annealing operations. IGA also converges faster, with fewer average generations than GA.

The results were analyzed to test the algorithms’ reliability and stability. Reliability is evaluated by the relative error rate, which compares the algorithm’s solution to previous studies [37,38,39]. Stability is assessed using the standard deviation of 20 runs to measure variability and consistency. The reliability and stability of both basic and improved genetic algorithms in solving the dataset are shown in Table 4. In terms of stability, the standard deviation of IGA results is smaller than that of GA. In terms of reliability, the relative error rate of IGA for solving all six datasets is significantly lower than that of GA. The results indicate that the IGA algorithm has better stability and reliability than GA.

**Figure 10:**
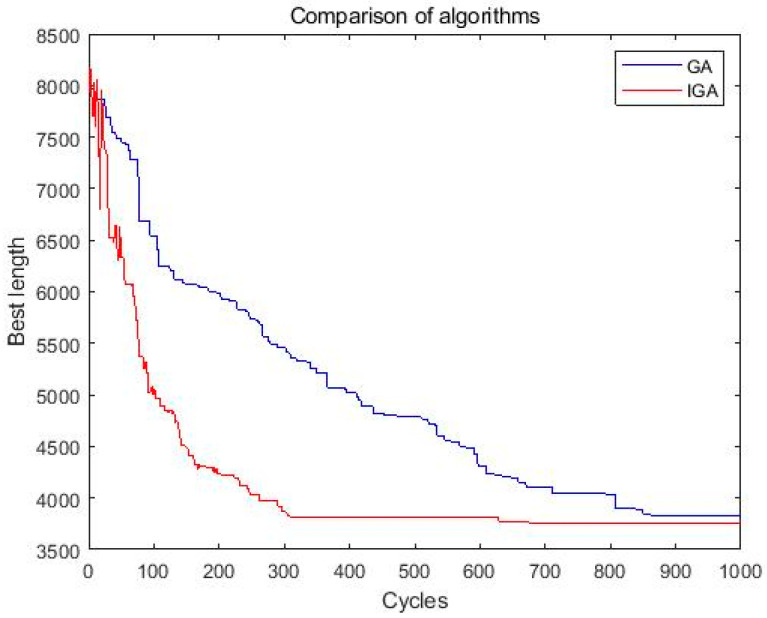
Comparison of IGA and GA (based on dataset Pr06).

Figure 10 clearly illustrates the convergence process of both the basic genetic algorithm (GA) and the improved genetic algorithm (IGA) when solving dataset Pr06. The comparison indicates that the IGA consistently achieves better convergence speed and optimization performance compared to the GA.

### 4.2. Case Study

The effectiveness of joint distribution in cold chain path optimization was verified through a practical application. Empirical data from three cold chain logistics companies in Beijing were used. These companies distribute the same frozen food products to customers in Beijing. Each operates independently on a small scale. The data represent China’s cold chain logistics industry. Table 5 shows customer locations, demand, and preferred time windows. In Table 5, negative integers correspond to depots. In joint distribution, companies integrate resources to serve all customers. The earliest depot departure is 6:00, and vehicles must return by 19:00. Average vehicle speeds within Beijing’s fifth ring were obtained from the Beijing Municipal Commission of Transportation. Data were collected from December 2020 to April 2021. Average speeds for each time period are shown in Table 6.

The parameters of the model are shown in Table 7: The number of distribution centers m is 3, and the number of all customer points n is 60. The fixed vehicle cost $C_{f}$ is CNY 150/vehicle, and the transportation cost $C_{t}$ is CNY 3/km. Punishment cost due to the early arrival $C_{e}$ and punishment cost due to the late arrival $C_{l}$ are respectively CNY 30/h and CNY 50/h. The cargo value $C_{p}$ is CNY 2000/ton, Deterioration factor of product freshness during transportation ε is 1, and the sensitivity factor of cold chain products θ is 0.002. The fuel consumption cost $C_{r}$ is CNY 6.7/L, the fuel consumption of the refrigeration equipment during vehicle driving $\alpha_{1}$ is 2 L/h, and the fuel consumption of the refrigeration equipment during loading and unloading $\alpha_{2}$ is 2.5 L/h. The carbon cost $C_{c}$ is CNY 0.25/kg, the carbon dioxide emission factor η is 2.63, and the load carbon emission factor β is $1.04\times 10^{-5}$.

#### 4.2.1. Comparison of Different Distribution Modes

**Figure 11:**
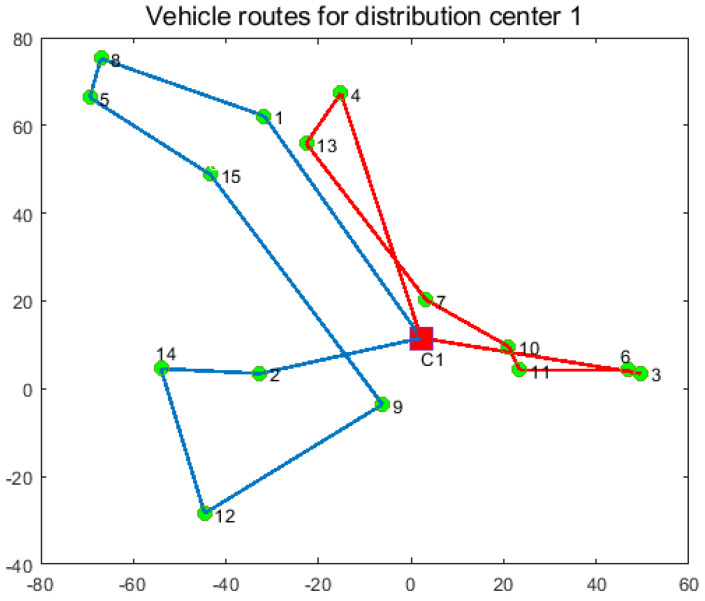
Optimized delivery routes for Distribution Center 1.

**Figure 12:**
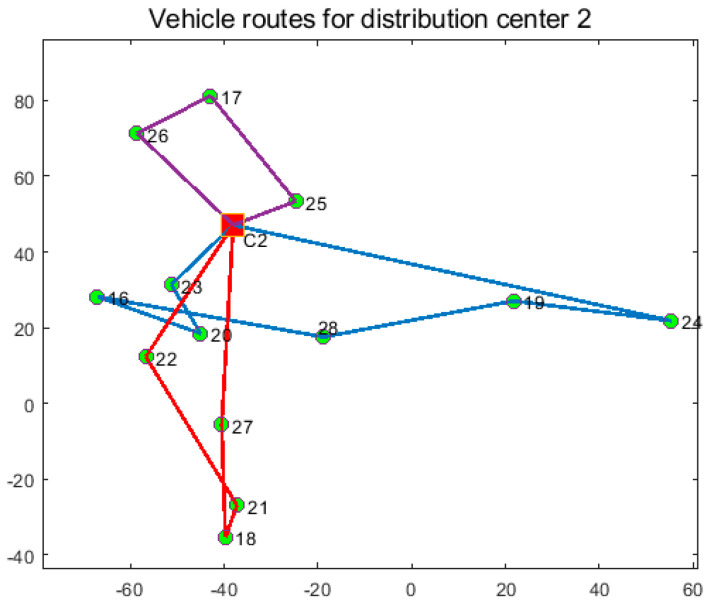
Optimized delivery routes for Distribution Center 2.

**Figure 13:**
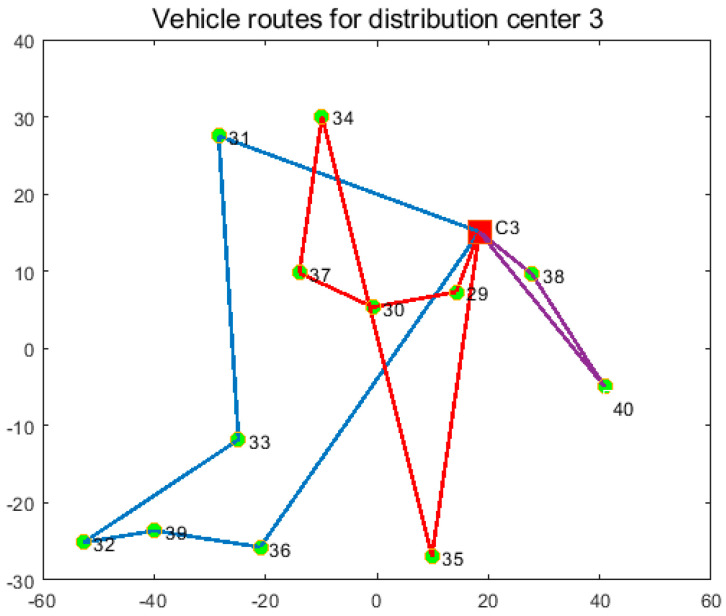
Optimized delivery routes for Distribution Center 3.

**Figure 14:**
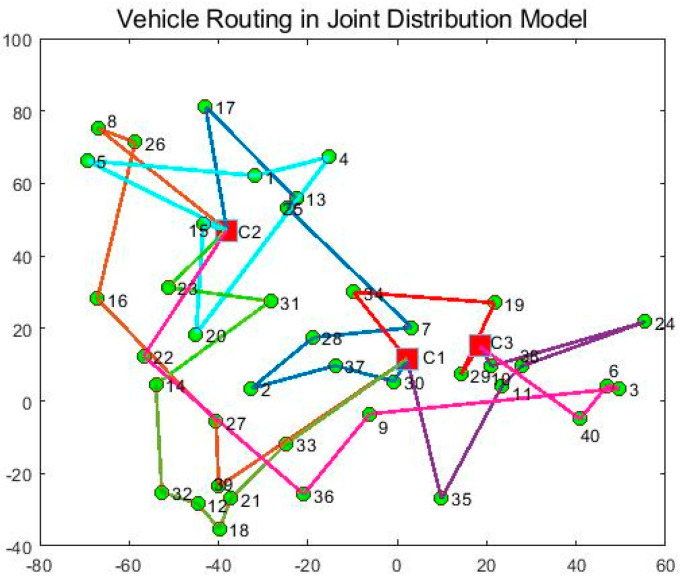
Optimized delivery routes for joint distribution mode.

In order to verify the effectiveness of the joint distribution mode, the joint distribution mode is compared with the independent distribution mode. In the single distribution mode, the three distribution centers distribute their respective customers, and the experimental results are shown in Table 8, Table 9 and Table 10 and Figure 11, Figure 12 and Figure 13. In the joint distribution mode, cold chain logistics companies share distribution centers and vehicles to serve customers together. The experimental results are shown in Table 11 and Figure 14.

From Figure 11, Figure 12, Figure 13 and Figure 14 and Table 8, Table 9, Table 10 and Table 11, the following can be observed: (1) Independent distribution has issues like roundabout routes, intersecting paths, and lower vehicle capacity due to scattered customers. Joint distribution coordinates resources, plans more efficient routes, reduces costs, and improves transportation efficiency. (2) Cold chain logistics companies can save 18.33% by adopting joint distribution, reducing carbon emissions by 15.8%. The CCLJDVRP-TPN model reduces fleet size by 12.5% and delivery distance by 19.88%. In conclusion, the model integrates storage and distribution resources using a sharing economy strategy, reducing costs and emissions, achieving economic and environmental benefits.

#### 4.2.2. Comparison of Different Congestion Speeds

According to Table 5, 6:00–8:00 and 17:00–19:00 are congestion periods. The model simulated vehicle speeds of 15 km/h, 20 km/h, and 25 km/h during these times. Table 12 shows the simulation results, reflecting how congestion affects vehicle routes and fleet size. The results show that joint distribution significantly reduces fleet size. Table 13 compares vehicle routes, demonstrating reduced fleet size and improved efficiency from optimized routes.

As shown in Table 12, increased vehicle speeds during congestion periods reduce total distribution costs, travel distances, carbon emissions, and penalty costs. (1) Regarding total distribution costs, increasing congestion speed from 15 km/h to 20 km/h reduces costs by 5.08%. Increasing speed further to 25 km/h reduces costs by 13.98%. (2) Vehicle travel distances at congestion speeds of 15 km/h, 20 km/h, and 25 km/h are 1344.81 km, 1238.07 km, and 1178.35 km, respectively. (3) In terms of carbon emissions, as vehicle speed increases, the distance traveled by the vehicle decreases and so do the carbon emissions. (4) Penalty costs decrease as vehicle speeds increase. The lowest penalty cost, 86.79, occurs at 25 km/h. Traffic congestion affects distribution costs, fuel consumption, carbon emissions, and customer service times. Increased congestion and reduced vehicle speeds lead to higher costs and emissions.

## 5. Discussion and Management Implications

In the present study, we propose a CCLJDVRP-TPN model considering time-varying road network. Joint distribution cooperation allows cold chain logistics companies to save costs, rent fewer vehicles, travel shorter distances, avoid congestion, and reduce carbon emissions. The government can promote sharing economy policies to incentivize participation in joint distribution. The discussion is as follows:

1. The CCLJDVRP-TPN model includes damage, refrigeration, and carbon costs, forming the total cold chain logistics cost. It accounts for congestion and vehicle speed variation, providing a practical solution. Experiments with empirical data show that resource sharing among cold chain enterprises achieves economic and environmental benefits.
2. Vehicle travel speed is crucial for optimizing cold chain product delivery. During traffic congestion, vehicle speed decreases, preventing the optimal path from being reached. This increases delivery time and delays timely service to customers.

Based on the above discussion, the following are some suggestions for cold chain logistics companies and governments.

3. Cold chain logistics enterprises must focus on reducing total distribution costs and improving logistics service quality to enhance their core competitiveness. In order to achieve these goals, joint distribution should be considered first. By collaborating and sharing resources such as warehouses, vehicles, and delivery schedules, cold chain logistics companies can reduce costs, emissions, and fleet size, improving efficiency and competitiveness. Ensuring product freshness and on-time delivery is crucial for quality. Companies must gather road network data, account for its time-varying nature, and improve route planning to ensure service quality. With growing awareness of sustainability, companies should also consider their carbon emissions. They should raise environmental awareness, cooperate with low-carbon policies, and build a positive business reputation.
4. The government plays a macro-control role, establishing a linkage mechanism between itself, industry associations, and leading enterprises. The government should oversee cold chain logistics planning based on industry analysis. It should promote sharing economy and joint distribution models, encouraging cooperation and platform building. Effective urban traffic planning can reduce congestion and improve distribution efficiency. Additionally, the government can collaborate with companies to develop low-energy equipment and technologies. This will promote the retrofitting and improvement of refrigeration systems, incentivizing cold chain logistics companies to reduce carbon emissions.

## 6. Conclusions

The present study constructs the CCLJDVRP-TPN model to address the joint problem of cold chain logistics under time-varying road network conditions. The model aims to minimize fixed costs, vehicle driving costs, penalty costs, refrigeration costs, loss costs, and carbon emission costs. An improved genetic algorithm was designed to solve the problem. The algorithm and the model’s validity were verified through comparison tests, leading to the following conclusions:

The improved genetic algorithm (IGA) integrates adaptive crossover and mutation probabilities with a simulated annealing strategy. This enhances global search capability and improves solution quality and stability. Comparison tests show that the IGA outperforms the basic genetic and simulated annealing algorithms in solution quality and convergence speed.

A case study of cold chain logistics distribution in Beijing was used to verify the effectiveness of the distribution strategy. The results show that the joint distribution strategy outperforms the individual model in total cost, vehicle travel distance, carbon emission, and fleet size. Vehicle path planning under different traffic congestion levels was compared. As congestion increases, total costs rise, and distribution efficiency decreases.

This study provides decision support methods for managers and the government. The results indicate that logistics companies should consider joint distribution strategies to serve customers together. They also need to account for traffic congestion and redesign distribution paths. The government can promote sharing economy policies to support logistics companies’ rapid development.

The present study has several limitations. First, it assumes that all customer information is static and known, excluding dynamic information such as new orders, order cancellations, delivery time changes, and other uncertainties. Additionally, the variation in efficiency among companies in joint distribution is not considered. Therefore, improving the profit of each company under the joint distribution model is a direction for future research.

## Figures and Tables

**Table 1:** Summary of representative literature and remaining gap.

No.	Study	Context	Cost Factors Includeda	Time-Varying Traffic Considered	Joint-Distribution/Resource-Sharing Considered
1	[12]	Perishable food VRP	Perishability	No	No
2	[13]	Fresh vegetable VRP	Multiple cost components	No	No
3	[15]	Portuguese food distribution	Multi-TW, vehicle types	No	No
4	[16]	Low-carbon cold-chain VRP	Refrigeration, carbon	No	No
5	[7]	Cold-chain VRP with carbon tax	Carbon, TW	No	No
6	[14]	Green MDVRP, shared resources	Fuel, penalty	Yes (time-dependent speed)	Partial (shared transport)
7	[22]	DVRP with congestion	Transport cost	Yes	No
8	[26]	MDVRP with delivery and pickup	Transport cost	No	Yes
9	[29]	Joint distribution with shared depots	Fuel consumption	No	Yes

**Table 2:** Description of symbols.

Symbols	Description
$C_{c}$	Carbon price
$C_{e}$	Punishment cost due to the early arrival
$C_{f}$	Fixed cost of each vehicle
$C_{l}$	Punishment cost due to the late arrival
$C_{p}$	Cold chain products’ price per unit
$C_{r}$	Refrigeration consumption cost per unit
$C_{t}$	Transportation cost of per unit distance
$d_{ij}$	Distance between nodes i and j
$g_{i}$	Demand for customer point i
i,j	Index of nodes (i,j=1,2,…,m,m+1,m+2,…,m+n)
K	Number of vehicles used
k	Index of vehicles (k=1,2,…,K)
m	Number of distribution centers (1,2,…,m)
n	Number of customers (m+1,m+2,…,m+n)
Q	The maximum load capacity of a vehicle
$Q_{ij}$	Products quantity from customer i to customer j
$S_{j}$	Service time of customer j
$T_{e}$	Time window’s starting time
$T_{l}$	Time window’s ending time
$T_{jk}$	Time point when vehicle k arrives at customer j
$t_{ij}$	Time of vehicle from node i to j
$v^{R}$	The vehicle travel speed in time period R
$W_{i}$	Time point from vehicle departure to customer i
$x_{ijk}$	0–1 value, when vehicle k delivers cargo from node i to node j, $x_{ijk}=1$; otherwise, $x_{ijk}=0$.
$\alpha_{1}$	The fuel consumption of refrigeration equipment per unit time during transportation
$\alpha_{2}$	The fuel consumption of refrigeration equipment per unit time during unloading
β	The load carbon emission factor
θ	Sensitivity factor for cold chain products
ε	Deterioration factor of product freshness during transportation
η	The coefficient values of the carbon emissions

**Table 3:** Results obtained using IGA and GA.

Datasets	IGA			GA		
	Optimal Value	Average Value	Average Number of Convergence Generations	Optimal Value	Average Value	Average Number of Convergence Generations
Pr01	1082.35	1086.21	70.8	1089.24	1132.72	84.37
Pr02	1763.07	1859.82	120.35	1806.32	1913.19	212.21
Pr03	2408.42	2501.01	203.56	2587.84	2712.56	321.31
Pr04	2852.29	2902.45	323.56	3183.85	3447.12	559.52
Pr05	3029.65	3388.55	545.07	3507.23	3795.35	691.79
Pr06	3758.36	3870.85	630.53	3834.35	4072.35	820.08

**Table 4:** Statistics of stability and reliability.

Datasets	Standard Solution	IGA			GA		
		Optimal Value	Relative Error Rate (%)	Standard Deviation	Optimal Value	Relative Error Rate (%)	Standard Deviation
Pr01	1074.12	1082.35	0.766	8.36	1089.24	1.407	10.76
Pr02	1762.21	1763.07	0.048	27.89	1806.32	2.503	33.09
Pr03	2373.65	2408.42	1.464	36.01	2587.84	9.023	62.38
Pr04	2815.48	2852.29	1.307	73.43	3183.85	13.083	98.58
Pr05	2965.18	3029.65	2.174	132.83	3507.23	18.280	171.54
Pr06	3612.72	3758.36	4.031	185.80	3834.35	6.134	230.90

**Table 5:** Customer locations, customer needs, and time window.

Serial Number	X (km)	Y (km)	Demands (t)	$T_{e}$	$T_{l}$	Service Time (h)	Affiliated Distribution Centers
−1	2.16	11.56	0	6:00	19:00		
−2	−38.12	47.1	0	6:00	19:00		
−3	18.39	15.11	0	6:00	19:00		
1	−31.73	62.14	1.3	12:20	18:24	0:25	−1
2	−32.66	3.46	0.9	7:10	10:25	0:18	−1
3	49.64	3.47	1.4	9:40	17:42	0:27	−1
4	−15.17	67.34	0.7	12:37	15:40	0:13	−1
5	−69.41	66.32	0.4	13:57	16:11	0:07	−1
6	46.91	4.27	0.7	6:40	11:18	0:13	−1
7	3.24	20.26	1.1	10:18	12:35	0:21	−1
8	−67	75.23	2.2	8:52	16:59	0:43	−1
9	−6.18	−3.57	1.1	10:46	13:21	0:21	−1
10	21.03	9.64	2	13:09	15:17	0:39	−1
11	23.48	4.29	0.9	9:42	11:43	0:18	−1
12	−44.62	−28.39	0.4	10:49	15:38	0:07	−1
13	−22.67	55.89	0.7	9:21	15:40	0:13	−1
14	−54.04	4.57	0.2	7:39	10:12	0:04	−1
15	−43.38	48.82	2.3	8:51	15:25	0:45	−1
16	−67.12	28.21	1.5	10:40	14:56	0:30	−2
17	−42.94	81.21	1.4	13:49	16:37	0:27	−2
18	−39.76	−35.33	2.3	11:02	18:56	0:45	−2
19	21.77	27.08	1.9	8:27	18:09	0:37	−2
20	−45.03	18.45	1.6	8:18	15:42	0:31	−2
21	−37.3	−26.9	2.1	13:27	14:59	0:41	−2
22	−56.76	12.37	1.6	14:04	18:01	0:31	−2
23	−51.33	31.37	0.4	7:12	11:01	0:07	−2
24	55.4	21.82	1.8	13:07	16:29	0:35	−2
25	−24.75	53.41	0.7	10:10	17:26	0:13	−2
26	−58.62	71.34	2.2	9:50	15:14	0:43	−2
27	−40.56	−5.7	1.1	11:20	14:04	0:21	−2
28	−18.78	17.54	1.2	11:47	14:49	0:24	−2
29	14.23	7.32	2	10:18	14:27	0:39	−3
30	−0.71	5.35	1.6	8:44	16:08	0:31	−3
31	−28.4	27.53	0.8	8:48	10:53	0:15	−3
32	−52.67	−25.13	1.7	11:30	17:00	0:33	−3
33	−24.83	−11.81	1.5	9:55	14:07	0:30	−3
34	−9.85	30.07	0.6	10:54	16:15	0:12	−3
35	9.88	−26.93	2.2	6:37	16:13	0:43	−3
36	−20.93	−25.73	2.4	7:37	15:07	0:47	−3
37	−13.92	9.76	0.5	6:46	12:39	0:10	−3
38	27.84	9.63	2.3	9:53	13:48	0:45	−3
39	−39.93	−23.61	2.3	12:18	18:54	0:45	−3
40	40.88	−4.97	0.8	9:40	12:45	0:15	−3

**Table 6:** Time-varying speed of Beijing.

Time Periods	Speed (km/h)
[6, 7]	25.6
[7, 8]	20.9
[8, 9]	26.52
[9, 10]	28.6
[10, 11]	29.6
[11, 12]	32.9
[12, 13]	30.5
[13, 14]	33.6
[14, 15]	30
[15, 16]	30
[16, 17]	28.8
[17, 18]	23.1
[18, 19]	20.9

**Table 7:** Parameter settings.

Symbols	Description
m	3
n	40
$C_{f}$	150
$C_{t}$	3
$C_{p}$	2000
$C_{r}$	6.7
$C_{e}$	30
$C_{l}$	50
$C_{c}$	0.25
ε	1
θ	0.002
$\alpha_{1}$	2
$\alpha_{2}$	2.5
η	2.63
β	$1.04\times 10^{-5}$
Q	10

**Table 8:** Optimized delivery routes for Distribution Center 1.

Vehicle	Distribution Route	Time of Vehicle Arrival at Customer’s Point
1	C1-2-14-12-9-15-5-8-1-C1	7:00-8:34-9:37-10:51-12:25-14:47-16:37-17:05-19:00-19:26
2	C1-3-6-11-10-7-13-4-C1	8:36-10:18-10:51-11:48-12:18-13:34-15:24-16:05-18:45

**Table 9:** Optimized delivery routes for Distribution Center 2.

Vehicle	Distribution Route	Time of Vehicle Arrival at Customer’s Point
1	C2-23-20-16-28-19-24-C2	7:00-7:59-8:39-10:02-12:05-13:46-15:32-19:00
2	C2-27-18-21-22-C2	10:28-12:08-13:25-14:28-16:39-18:59
3	C2-26-17-25-C2	9:19-10:24-11:42-13:13-13:54

**Table 10:** Optimized delivery routes for Distribution Center 3.

Vehicle	Distribution Route	Time of Vehicle Arrival at Customer’s Point
1	C3-31-33-32-39-36-C3	6:59-9:02-10:39-12:06-13:04-14:27-17:13
2	C3-29-30-37-34-35-C3	10:09-10:27-11:34-12:34-13:22-15:32-17:56
3	C3-38-40-C3	9:42-10:05-11:27-12:41

**Table 11:** Optimized delivery routes for joint distribution mode.

Vehicle	Distribution Route	Time of Vehicle Arrival at Customer’s Point
1	C1-30-37-2-28-7-25-13-17-C2	8:24-8:40-9:41-10:31-11:26-12:33-14:14-14:35-15:53-17:41
2	C2-8-26-16-27-39-C1	7:00-8:44-9:47-11:54-13:44-14:42-17:25
3	C1-35-11-38-24-10-C3	6:37-8:20-10:15-10:47-12:29-14:11-15:03
4	C2-23-31-14-32-12-18-21-33-C1	6:59-7:59-8:59-10:26-11:28-12:18-12:43-13:44-15:05-16:48
5	C3-40-6-3-9-36-22-C2	9:10-10:13-10:51-11:10-13:24-14:37-17:15-19:00
6	C2-15-20-4-1-5-C2	8:35-8:48-10:36-12:56-13:41-15:23-16:46
7	C3-29-19-34-C1	10:09-10:27-11:45-13:23-14:16

**Table 12:** Comparison between single and joint distribution mode.

Distribution Mode	Total Cost	Total Distance	Carbon Emissions	Fleet Size
Single distribution	9190.31	1541.504	376.936	8
Joint distribution	7505.78	1235.005	317.304	7
Rate of decline	18.33%	19.88%	15.8%	12.5%

**Table 13:** Comparison of vehicle routes with different congestion speeds.

Speed (km/h)	Total Cost	Total Distance	Carbon Emissions	Time Penalty Cost
15	8295.21	1344.81	352.89	227.156
20	7876.54	1238.07	338.01	225.41
25	7135.56	1178.35	306.68	86.79

## Data Availability

The authors will supply the relevant data in response to reasonable requests.
